# Asherman Syndrome in Mexican Women: Clinical Characteristics, Management, and Outcomes at a Tertiary Hospital

**DOI:** 10.3390/jcm15103672

**Published:** 2026-05-10

**Authors:** Andrea Olguín-Ortega, Jessica Aidee Mora-Galván, Fernando Escobar-Ponce, Fernanda Villalobos-Mendoza, Alejandro Rendón-Molina, Oliver Cruz-Orozco, Enrique Reyes-Muñoz

**Affiliations:** 1 Department of Gynecology, National Institute of Perinatology, Mexico City 11000, Mexico; dramoragalvan@gmail.com (J.A.M.-G.); drescobarponce@gmail.com (F.E.-P.); fernandavillmen@gmail.com (F.V.-M.); oliverpco@gmail.com (O.C.-O.); 2 Division of Gynecology and Human Reproduction, National Institute of Perinatology, Mexico City 11000, Mexico; arendonm.87@gmail.com; 3 Coordination of Gynecological and Perinatal Endocrinology, National Institute of Perinatology, Mexico City 11000, Mexico

**Keywords:** Asherman syndrome, intrauterine adhesions, intrauterine synechiae, gynatresia

## Abstract

**Background/Objective**: Asherman syndrome is an acquired intrauterine adhesive disorder associated with menstrual abnormalities, infertility, recurrent pregnancy loss, and adverse reproductive outcomes. Data from Latin American tertiary referral centers remain limited. To characterize clinical history, classification, hysteroscopic management strategies, and anatomical and reproductive outcomes in a single cohort of Mexican women with Asherman syndrome. **Methods**: This retrospective cohort study included women identified through institutional electronic records between July 2016 and December 2023 with a diagnosis of Asherman syndrome and analyzable hysteroscopic records. Women were followed for twelve months. Recurrence was defined as hysteroscopic evidence of intrauterine adhesions during follow-up. Among women with follow-up hysteroscopy, two-sided Fisher’s exact tests were used to calculate odds ratios and 95% confidence intervals. **Results**: Fifty-four women were analyzed. A prior uterine procedure was documented in 44 women (81.5%), with sharp curettage in 35 (64.8%). The most common reasons for consultation were secondary infertility (29.6%), abnormal uterine bleeding (27.8%), primary infertility (20.4%), and recurrent pregnancy loss (13.0%). Disease severity was classified as mild in 30 women (55.6%), moderate in 11 (20.4%), and severe in 7 (13.0%). Hysteroscopic intervention was predominantly performed with cold knife adhesiolysis (83.3%). Twelve-month follow-up hysteroscopy was performed in 38 women (70.4%); recurrence was identified in 30 (55.6%). Among the 34 women with reproductive intent, 12 achieved a live birth, corresponding to a live birth rate of 35.3%. **Conclusions**: Prior uterine instrumentation, particularly sharp curettage, was the most frequent antecedent. Recurrence remained common despite surgical management, highlighting the need for standardized postoperative surveillance and preventive strategies.

## 1. Introduction

Asherman syndrome is a rare, acquired condition characterized by obliteration of the uterine cavity due to partial or complete fibrous intrauterine adhesions [1]. In developed countries, it is predominantly caused by prior intrauterine surgical trauma and is often asymptomatic, though it may present with hypo- or amenorrhea and contribute to infertility and adverse pregnancy outcomes [2]. Endometrial injury may heal through a nonregenerative process in which normal tissue is replaced by fibrous connective tissue, promoting adhesion between opposing endometrial surfaces and leading to partial or complete obliteration of the uterine cavity and/or cervical canal [3]. The adhesions may involve the endometrium, myometrium, or multiple layers of connective tissue [3,4].

The precise pathophysiological mechanism of Asherman syndrome remains uncertain. However, pregnancy history is the most commonly cited risk factor preceding Asherman syndrome [3,4,5,6]. In some case analyses, abortion/miscarriage curettage (66.7%) and postpartum curettage (21.5%) were the two most significant factors associated with Asherman syndrome [4,5,6]. Infectious etiologies must also be considered, as endometrial tuberculosis may account for up to 23.7% of cases in underdeveloped countries, underscoring its continued relevance as a cause of Asherman syndrome in low-resource settings [4]. Other etiologic factors reported include intrauterine devices, myomectomy, uterine surgery such as cesarean sections, diagnostic curettage, and hysteroscopic surgery [4,5,6].

Although the exact prevalence of Asherman syndrome is unknown, it varies between 1.5% in cases of infertility and 30% following intrauterine instrumentation [4].

Enhanced recognition of the symptoms of intrauterine adhesive disease, along with an understanding of its common etiologies and preceding events, is essential for timely diagnosis, effective patient counseling, and appropriate management [5]. Hysteroscopy remains the gold standard for diagnosis, and treatment consists of hysteroscopic adhesiolysis to restore normal uterine cavity anatomy [2]. Treatment of Asherman syndrome can be particularly challenging in severe cases, as it aims to restore the normal architecture of the uterine cavity, reestablish normal menstruation, improve fertility outcomes, and reduce the risk of miscarriage [6].

Primary prevention of intrauterine adhesions involves meticulous surgical techniques and the use of barrier strategies, including gels, intrauterine devices or balloons, and hyaluronate–carboxymethylcellulose or polyethylene oxide–sodium carboxymethylcellulose membranes as anti-adhesive agents [7]. Although hysteroscopic adhesiolysis is the gold standard, its success is limited by high recurrence rates of adhesions and associated obstetrical risks [8]. Several studies have reported that hormonal therapy after restoration results in a significantly higher rate of return to normal menstruation [9,10]. However, the absence of a definitive optimal therapy highlights the need for further research [11]. Evidence from Latin American tertiary referral settings remains limited. The present study aimed to describe the clinical presentation, prior uterine procedures, hysteroscopic management, recurrence, and reproductive outcomes among women treated for Asherman syndrome at the National Institute of Perinatology in Mexico City between July 2016 and December 2023.

## 2. Materials and Methods

### 2.1. Design and Participants

A retrospective, observational, single-cohort study was conducted to assess clinical history, classification, management, and reproductive outcomes among Mexican women diagnosed with Asherman syndrome at the National Institute of Perinatology in Mexico City from July 2016 to December 2023. The study protocol was reviewed and approved by the Institutional Ethics and Research Internal Review Board (Registry number: CEI-RETRO-01-2025; approval date: January 2025).

Data were obtained from electronic medical records. Potential cases were identified using predefined search terms, including Asherman syndrome, intrauterine adhesions, gynatresia, and intrauterine synechiae. Eligibility criteria included women with one or more search terms and or diagnosis of Asherman syndrome established by imaging techniques such as hysterosalpingography, sonohysterography, or transvaginal ultrasound. Only women with intrauterine adhesions confirmed by initial hysteroscopy were included. Women with suspected Asherman syndrome without evidence of intrauterine adhesions on hysteroscopy were excluded. A total of 54 women met the inclusion criteria and were included in the analysis. Collected variables included demographic characteristics, relevant clinical history, diagnostic findings, therapeutic interventions, recurrence, and reproductive outcomes.

During the preparation of this manuscript, the authors used ChatGPT (GPT-5, OpenAI, San Francisco, CA, USA; GPT-5 model accessed via the ChatGPT interface, 24 April 2026) for English-language editing, grammar correction, and academic style refinement and manuscript formatting. Google Colaboratory (Google LLC, Mountain View, CA, USA; model accessed 24 April 2026) was used to assist in generating and preparing the graphical abstract. The authors have reviewed and edited the outputs and take full responsibility for the content of this publication.

### 2.2. Procedure

Women included underwent office hysteroscopy with a Karl Storz Bettocchi hysteroscope (Karl Storz SE & Co. KG, Tuttlingen, Germany) with an outer sheath, performed under a vaginoscopic (no-touch) approach that avoids a speculum and tenaculum, thereby reducing patient discomfort and cervical manipulation. Prophylactic antibiotics consisted of a single 1 g oral dose of azithromycin taken the night before the procedure. For analgesia, 30 mg of sublingual ketorolac was administered 30 min prior to the procedure unless contraindicated. The examination was conducted in an outpatient setting without anesthesia. Normal saline was used as the distension medium. The uterine cavity was systematically evaluated, including inspection of the endocervical canal, endometrial lining, uterine walls, tubal ostia, and any intrauterine pathology. Findings were recorded in real time via video documentation and registered using a standardized hysteroscopy reporting form that includes a diagrammatic representation of the uterine cavity to annotate the location and nature of observed lesions or anomalies. All women were followed at the hospital for at least 12 months to monitor their clinical course, assess changes in menstrual patterns, and determine whether they achieved pregnancy.

### 2.3. Clinical History

The variables of the clinical history were: (1) Conservative uterine surgery includes various organ-preserving procedures such as excision or cytoreductive surgery for endometriosis and adenomyosis, reconstructive techniques for placenta accreta spectrum and uterine incisional necrosis, and, in select cases, fertility-sparing treatment of early-stage endometrial cancer [12]. (2) The history of abdominal myomectomy is defined as the removal of fibroids followed by the surgical restoration of normal uterine anatomy [13]. (3) Hysteroscopic myomectomy involves removing submucosal myomas either by enucleation or slicing technique; the former involves dissecting the fibroid along the pseudocapsule with minimal thermal injury, while the latter fragments the submucosal and intramural components, resulting in greater resection but also a higher risk of thermal damage and intravasation [14]. (4) Dilation and curettage involve dilating the cervix and scraping the endometrial lining, and it may serve as a diagnostic or therapeutic procedure for abnormal uterine bleeding or early pregnancy loss [15].

### 2.4. Outcomes After Hysteroscopic Management

The outcomes after initial treatment were defined as follows; (a) Recurrence: reformation of intrauterine adhesions or endometrial scarring after 12 months of initial treatment, potentially resulting in persistent partial or complete endometrial dysfunction [16]; (b) Live birth: the complete expulsion or extraction of a fetus from the mother, regardless of gestational age, that shows any signs of life, such as heartbeat, breathing, or voluntary muscle movement, resulting in a newborn infant [17]; (c) Infertility: a clinical condition marked by a reduced or absent ability to conceive after a specified period of regular, unprotected sexual intercourse [18]; (d) Reproductive intent: the desire to conceive following hysteroscopic management, including women with primary or secondary infertility, as well as those with a history of recurrent pregnancy loss.

### 2.5. Statistical Analysis

Qualitative variables were reported as frequencies and proportions. Quantitative variables were reported using means and standard deviations and/or medians and interquartile ranges, depending on each variable’s distribution. Normality was assessed using the Shapiro–Wilk test and visual inspection of histograms and Q–Q plots. Exploratory comparisons for recurrence were restricted to women with follow-up hysteroscopy. Two-sided Fisher’s exact tests were used because of the small sample size, and odds ratios with 95% confidence intervals were calculated. When a 2 × 2 table contained a zero cell, the Haldane–Anscombe correction was applied. A *p*-value < 0.05 was considered statistically significant. Analyses were performed using IBM SPSS Statistics for Windows, version 24.0 (IBM Corp., Armonk, NY, USA). A multivariable logistic regression analysis was performed; this analysis was considered exploratory.

## 3. Results

**Figure 1:**
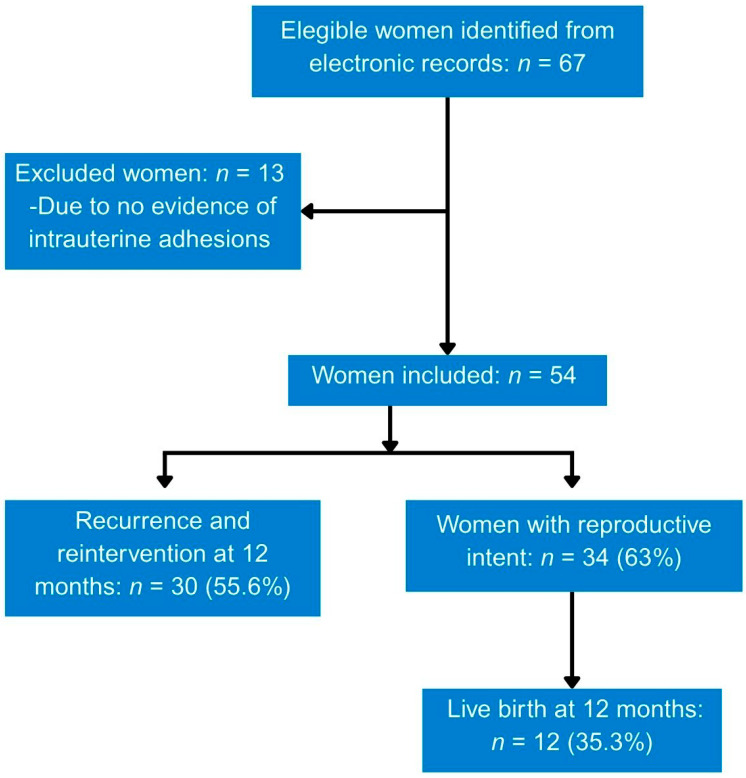
Flow diagram of women with Asherman syndrome included in the study.

From July 2016 to December 2023, 67 women were eligible based on one or more search terms in the electronic record and/or a diagnosis of Asherman syndrome on imaging studies. Of these, 13 were excluded because hysteroscopy showed no evidence of intrauterine adhesions, leaving 54 who met the inclusion criteria. Women with Asherman syndrome were followed for at least 12 months after the initial hysteroscopy (Figure 1).

### 3.1. Demographic and Obstetric Characteristics

Demographic characteristics and obstetric history were collected and displayed in Table 1. The mean age was 38.7 ± 6.3 years. BMI showed a non-normal distribution with a median of 25.9 kg/m^2^ (IQR, 24.2–29.8).

### 3.2. History of Uterine Procedures

At least one previous uterine procedure was documented in 44 women (81.5%). The most frequent antecedent was sharp curettage, reported in 35 women (64.8%), followed by prior hysteroscopy in 10 (18.5%) and open myomectomy with cavity entry in 9 (16.7%) (Table 2). Percentages do not add to 100% because several women had more than one previous procedure.

### 3.3. Clinical Presentation, Imaging, and Initial Hysteroscopic Findings

The most common reason for consultation was secondary infertility (29.6%), followed by abnormal uterine bleeding (27.8%), primary infertility (20.4%), and recurrent pregnancy loss (13.0%). A preoperative imaging study suggestive of synachiae was documented in 23 women (42.6%). At initial hysteroscopy, intrauterine synachiae were observed in all women; other findings included endometrial polyps, myomas, uterine septum, and distorted uterine cavity (Table 3).

According to the American Society for Reproductive Medicine (ASRM) classification [19], the severity of Asherman syndrome was mild in 30 women (55.6%), moderate in 11 (20.4%), and severe in 7 (13.0%). Six cases (11.1%) were not classifiable because the information required for formal grading was incomplete (Table 4).

### 3.4. Management and Outcomes

Hysteroscopic cold knife adhesiolysis was the most common approach, performed in 45 women (83.3%). Postoperative recurrence-prevention strategies were documented, including intrauterine device or mechanical barrier use (20 [37.0%]), hormonal therapy (11 [20.4%]), and hyaluronic acid (8 [14.8%]). Percentages are calculated using the full analytic cohort as the denominator and do not sum to 100% because the categories are not mutually exclusive (Table 5). Intrauterine adhesion recurrence was identified in 30 women (55.6%). Live birth occurred in 12 of 34 women with reproductive intent (35.3%).

### 3.5. Exploratory Analysis of Recurrence

Exploratory recurrence analyses were limited to the 38 women who had follow-up hysteroscopy at 12 months. The remaining women became pregnant or had regular menstrual cycles. Moderate/severe ASRM classification was associated with recurrence (OR 23.22, 95% CI 1.24–436.39; *p* = 0.005). A history of sharp curettage was also associated with recurrence (OR 6.29, 95% CI 1.24–31.96; *p* = 0.040). Postoperative preventive measures were not significantly associated with recurrence (OR 0.55, 95% CI 0.13–2.21; *p* = 0.403) (Table 6).

## 4. Discussion

This retrospective single-cohort study at a tertiary referral center describes the clinical profile, prior uterine procedures, management, and outcomes of 54 Mexican women evaluated for Asherman syndrome with analyzable hysteroscopic data. Notably, prior uterine instrumentation was common, with at least one prior uterine procedure documented in more than four-fifths of women. In the exploratory subgroup analysis, sharp curettage was the most common procedure and was associated with recurrence, which occurred more frequently in our cohort than reported in the literature (67.3%) [20,21]. This finding supports the clinical relevance of minimizing basal endometrial trauma when uterine evacuation or intrauterine surgery is required. Although intrauterine adhesion formation is one possible consequence of endometrial injury, uterine evacuation procedures may also be associated with other delayed complications. De Cicco et al. reported a rare case of delayed uterine perforation with small bowel incarceration diagnosed 11 months after dilatation and curettage performed for retained membranes. Transvaginal ultrasound identified bowel entering the uterine cavity, and laparoscopy confirmed intestinal prolapse through a uterine defect [22]. Similarly, Stabile et al. systematically reviewed fallopian tube incarceration or intussusception following vacuum aspiration or dilatation and curettage [23]. Together, these reports reinforce the importance of careful surgical technique, detailed clinical history, appropriate imaging, and endoscopic evaluation, when clinically indicated, in women with a history of uterine instrumentation.

Another important factor was open myomectomy with cavity entry, although in this simple cohort the percentage was lower than reported in other studies. In Bhandari’s study, the incidence of post-myomectomy intrauterine adhesions was 21.5%, regardless of fibroid features and surgical technique [24]. The main reason women sought medical attention for Asherman syndrome in this study was secondary infertility, which accounted for 33% of cases. This suggests that Asherman syndrome should be suspected in women with infertility and a history of uterine surgery. A systematic review found that an absent or non-functioning uterus (uterine factor infertility) is a major contributor to female infertility and first quantified its global prevalence as ranging from 2.1% to 16.7% of cases [25].

The most common clinical presentations were secondary infertility, abnormal uterine bleeding, primary infertility, and recurrent pregnancy loss. In a similar study, amenorrhea was a strong indicator of Asherman syndrome (OR = 26.19) [26]. These findings emphasize that Asherman syndrome should be considered in women with reproductive dysfunction or menstrual changes after prior intrauterine procedures.

Preoperative imaging identified adhesions in fewer than half of the cohort, whereas hysteroscopy allowed direct confirmation and grading of intrauterine findings. As reported by others, for initial evaluation, less invasive modalities such as contrast sonohysterography or hysterosalpingography may be useful; however, definitive diagnosis relies on hysteroscopy [27]. The study therefore supports the central role of hysteroscopy in diagnosis and management.

Hysteroscopy also enabled grading of disease severity according to the ASRM classification. Mild disease was more common in our series than in the comparator study (55% vs. 40%) [28], which may reflect differences in referral patterns, diagnostic timing, or classification criteria. Cold knife hysteroscopic adhesiolysis was the most commonly used technique, accounting for 83.3%. This preference reflects its feasibility in the outpatient setting. However, in another series, electrosurgical adhesiolysis for Asherman syndrome in infertile patients did not appear to increase the need for repeat hysteroscopic adhesiolysis or adversely affect endometrial thickness compared with cold knife hysteroscopic adhesiolysis [29].

After adhesiolysis, 42.6% of women received measures to prevent recurrence, including an intrauterine device (IUD) in 37%. In a report of 43 women treated for Asherman syndrome, all received hyaluronic acid, a copper IUD, and estrogen therapy; among 38 women trying to conceive, the conception rate was 82%, and the live birth rate was 63% [30]. The second most common preventive method was oral estrogen therapy (20.4%), but because doses and treatment durations varied, the literature lacks robust evidence to recommend it for preventing recurrence. In a cohort of 114 women, postoperative estrogen therapy was not associated with reduced adhesion recurrence or improved reproductive outcomes, as recurrence, pregnancy, and live birth rates were similar between women who received estrogen and those who did not [31]. Although intrauterine devices, hormonal therapy, and hyaluronic acid were used to prevent recurrence, none showed a statistically significant protective association in the exploratory analysis. This does not prove lack of efficacy; rather, it reflects limited sample size, non-random treatment allocation, and likely confounding by indication, as more severe cases may have been more likely to receive adjuvant strategies.

Follow-up hysteroscopy was performed in 70.4% of women at 12 months, with an average interval of 3 months after the initial procedure, roughly 1 month later than the timing recommended by international guidelines to reduce recurrence rates [32]. However, in this simple retrospective cohort, the recurrence and hysteroscopic reintervention rates were higher than reported in the literature (55%) [5]. In another study, women with severe intrauterine adhesions who underwent hysteroscopic cold knife adhesiolysis combined with sequential high-dose estrogen and progesterone therapy had lower recurrence rates and higher treatment effectiveness than with surgery alone (94.07% vs. 79.27%) [33]. Recurrences occurred predominantly in women classified as having moderate/severe disease according to the ASRM criteria. This finding suggests that advanced forms of Asherman syndrome may be a risk factor for recurrence. This result was influenced by heterogeneity in preventive strategies, variability in postoperative follow-up, and structural constraints inherent to real-world tertiary public care settings.

Live birth occurred in 35.3% of women with reproductive intent. In contrast, a previous study reported an overall take-home newborn rate of 67.4% after adhesiolysis, with better outcomes among younger women, those who underwent first-trimester procedures before Asherman syndrome, those with lower-grade adhesions, and those without a miscarriage after treatment [34].

This study has several limitations. It was a retrospective, single-center study based on electronic record search terms, which may have missed cases due to coding or terminology variability. The study was descriptive and lacked an external control group; therefore, previous procedures should be interpreted as antecedents rather than definitive risk factors. Imaging operator experience was not consistently recorded. Follow-up hysteroscopy was not performed in all women, and loss to follow-up was substantial. Finally, the small sample size limited multivariable modeling and reduced the precision of the odds ratios, as reflected by wide confidence intervals. Multivariable regression was considered exploratory and should not be interpreted as confirmatory.

## 5. Conclusions

In this single cohort of Mexican women with Asherman syndrome, prior uterine instrumentation—particularly sharp curettage—was the most common antecedent among those evaluated for Asherman syndrome. Hysteroscopy was essential for confirmation, classification, treatment, and surveillance. Recurrence was common among women who underwent follow-up hysteroscopy, especially among those with moderate or severe disease. These findings support the need for meticulous endometrial-sparing techniques, standardized postoperative follow-up, and more rigorous prospective assessment of interventions to prevent recurrence.

## Figures and Tables

**Table 1:** Demographic characteristics and obstetric history of a cohort of Mexican women with Asherman syndrome.

Variable	Asherman Syndrome (*n* = 54)	Range
Age, years	38.7 ± 6.3	23–52
BMI, kg/m^2^	25.9 (24.2–29.8)	21.8–40.2
Pregnancies	1.5 (1.0–3.0)	0–6
Vaginal deliveries	0 (0–0)	0–1
Cesarean deliveries	0 (0–0)	0–2
Miscarriages	1 (0–3)	0–5
Ectopic pregnancies	0 (0–0)	0–1

Data are presented as mean ± standard deviation and/or median and (interquartile range).

**Table 2:** History of uterine procedures in Mexican women with Asherman syndrome.

Procedure	Frequency and (%) *n* = 54
Any previous uterine procedure	44 (81.5)
Sharp curettage	35 (64.8)
Vacuum aspiration	4 (7.4)
Prior hysteroscopy	10 (18.5)
Open myomectomy with cavity entry	9 (16.7)
Open myomectomy without cavity entry	3 (5.6)
Laparoscopic myomectomy with cavity entry	1 (1.9)
Laparoscopic myomectomy without cavity entry	2 (3.7)
Hysteroscopic myomectomy	3 (5.6)
Septoplasty	3 (5.6)
Polypectomy	1 (1.9)

**Table 3:** Clinical presentation, imaging, and initial hysteroscopic findings among Mexican women with Asherman syndrome.

Variable	*n* = 54 (%)
Secondary amenorrhea	4 (7.4)
Primary infertility	11 (20.4)
Secondary infertility	16 (29.6)
Dysmenorrhea	1 (1.9)
Recurrent pregnancy loss	7 (13.0)
Abnormal uterine bleeding	15 (27.8)
Two-dimensional ultrasound suggestive of adhesions	8 (14.8)
Sonohysterography suggestive of adhesions	15 (27.8)
Hysterosalpingography suggestive of adhesions	8 (14.8)
Any imaging study suggestive of adhesions	23 (42.6)
Endometrial polyp on initial hysteroscopy	13 (24.1)
Myoma on initial hysteroscopy	6 (11.1)
Uterine septum on initial hysteroscopy	5 (9.3)

Percentages are calculated using the full analytic cohort as the denominator and do not add to 100% because categories are not mutually exclusive.

**Table 4:** American Society for Reproductive Medicine classification at initial hysteroscopy among Mexican women with Asherman syndrome (*n* = 54).

ASRM Category	*n* = 54 (%)
Mild	30 (55.6)
Moderate	11 (20.4)
Severe	7 (13.0)
Not classifiable	6 (11.1)

**Table 5:** Hysteroscopic management, recurrence-prevention strategies, and reproductive outcomes among Mexican women with Asherman syndrome.

Variable	*n* (%)
Cold-knife adhesiolysis	45 (83.3)
Energy-based resection	1 (1.9)
Intrauterine device/mechanical barrier	20 (37.0)
Hormonal therapy	11 (20.4)
Hyaluronic acid	8 (14.8)
Twelve-month follow-up hysteroscopy	38 (70.4)
Recurrence of intrauterine adhesions	30 (55.6)
Reintervention among women with follow-up hysteroscopy	30 (55.6)
Live birth, total cohort	12 (22.2)
Live birth among women with reproductive intent	12 (35.3)

**Table 6:** Exploratory associations with Asherman syndrome recurrence among Mexican women with hysteroscopic follow-up (*n* = 38).

Variable	Recurrence in Exposed, *n*/*N* (%)	Recurrence in Unexposed, *n*/*N* (%)	OR (95% CI)	*p*
History of sharp curettage	22/25 (88.0)	7/13 (53.8)	6.29 (1.24–31.96)	0.040
Hormonal therapy	9/9 (100.0)	20/29 (69.0)	8.80 (0.46–167.57)	0.082
Intrauterine device/mechanical barrier	15/18 (83.3)	14/20 (70.0)	2.14 (0.45–10.26)	0.454
Hyaluronic acid	8/8 (100.0)	21/30 (70.0)	7.51 (0.39–143.90)	0.159
Any preventive strategy	16/19 (84.2)	13/19 (68.4)	2.46 (0.51–11.80)	0.447
Moderate/severe ASRM classification	16/16 (100.0)	13/22 (59.1)	23.22 (1.24–436.39)	0.005

Two-sided Fisher’s exact test. Odds ratios and 95% confidence intervals were calculated from 2 × 2 tables; the Haldane–Anscombe correction was applied when a zero cell was present. CI, confidence interval; OR, odds ratio.

## Data Availability

Please contact the corresponding author for data requests.

## Abbreviations

The following abbreviations are used in this manuscript:

ASRM	American Society for Reproductive Medicine
BMI	Body Mass Index
IQR	Interquartile Range
IUD	Intrauterine Device
IRB	Institutional Review Board
COVID-19	Coronavirus Disease 2019
2D	Two-Dimensional
